# Wrapping Effects within a Proposed Function-Rescue Strategy for the Y220C Oncogenic Mutation of Protein p53

**DOI:** 10.1371/journal.pone.0055123

**Published:** 2013-01-24

**Authors:** Sebastián R. Accordino, J. Ariel Rodríguez Fris, Gustavo A. Appignanesi

**Affiliations:** Sección Fisicoquímica, INQUISUR-UNS-CONICET and Departamento de Química, Universidad Nacional del Sur, Bahía Blanca, Argentina; Instituto de Tecnologica Química e Biológica, UNL, Portugal

## Abstract

Soluble proteins must protect their structural integrity from water attack by wrapping interactions which imply the clustering of nonpolar residues around the backbone hydrogen bonds. Thus, poorly wrapped hydrogen bonds constitute defects which have been identified as promoters of protein associations since they favor the removal of hydrating molecules. More specifically, a recent study of our group has shown that wrapping interactions allow the successful identification of protein binding hot spots. Additionally, we have also shown that drugs disruptive of protein-protein interfaces tend to mimic the wrapping behavior of the protein they replace. Within this context, in this work we study wrapping three body interactions related to the oncogenic Y220C mutation of the tumor suppressor protein p53. Our computational results rationalize the oncogenic nature of the Y220C mutation, explain the binding of a drug-like molecule already designed to restore the function of p53 and provide clues to help improve this function-rescue strategy and to apply in other drug design or re-engineering techniques.

## Introduction

The tumor suppressor protein p53 participates in most of the cases of human cancer and is mutated approximately in half of them [1]. In turn, in almost one third of such mutations the activity loss is simply a consequence of a lowering in the melting temperature of the core DNA-binding domain of p53 [2]–[4] that leads to a quickly denaturation in the cell [5] (in fact, the wild-type p53 molecule, that is, the protein without mutations, has very low stability itself [1]–[7]). The Y220C mutation, a mutation that significantly lowers the thermodynamic stability of the protein, is one of the most frequent oncogenic mutations in p53. The mutation site, located at the protein’s surface, is distant from the DNA binding site of p53 and does not alter the overall structure of such region [6]. Interestingly, such mutation of a tyrosine to a cysteine promotes the formation of a potentially druggable cavity at the mutation site. Thus, the binding of a small molecule at this cavity could stabilize the mutant protein without affecting the function of p53 and also leaving aside wild-type p53 molecules. This insight has been exploited by the group of Alan Fersht in a series of beautiful papers [2]–[7]. Such group discovered a drug-like molecule called PhiKan083, a carbazole derivative that binds to the mutational cavity of p53-Y220C [7], with moderate affinity (K_d_ around 150 µM), raising the melting temperature and slowing down the rate of denaturation of p53-Y220C. Additionally, fragment screening and isopropanol solvation studies were conducted in order to map the different interaction sites or “hot spots” of such mutational cavity [2] in order to provide a blueprint for the design of molecules that bind to p53-Y220C with drug-like (submicromolar) affinity.

In turn, the introduction of the wrapping concept by Prof. Ariel Fernández has thrown a new light onto the protein stability and protein binding fields [8]–[16]. To prevail in water environments, soluble proteins must protect their backbone hydrogen bonds (HBs) from the disruptive effect of water attack by clustering nonpolar residues around them [8]–[16]. This exclusion of surrounding water, or wrapping effect, also enhances the electrostatic contribution by modulating the local dielectric (de-screening the partial charges) and thus stabilizes the HB. Thus, underwrapped interactions, called dehydrons, represent thermodynamically unstable, vulnerable sites [8], [16] and the level of underwrapping has been shown to correlate with the degree of structural disorder [13] (in fact, the most extreme case is represented by the prion proteins which, being the worst wrapped proteins in the Protein Data Bank, are so thermodynamically unstable in monomeric form in solution that they aggregate to form amyloids [8]; also among the worst wrapped proteins we can find the toxin proteins, whose dehydrons-rich structure can only be sustained by the establishment of disulfide bonds [8]). A main notion that derives from this picture is that dehydrons are adhesive [8], [9], hence promoters of molecular associations because their inherent stability increases upon approach of additional nonpolar groups. Thus, dehydrons constitute key motifs that signal protein binding sites. Under this scenario, the integrity of the interface of a biomolecular complex (when a protein binds to another protein or to a small-molecule ligand) becomes extremely reliant on intermolecular cooperativity based on three-body correlations [8]–[16]: a third nonpolar body protects an electrostatic interaction pairing the other two and not all three bodies belong to the same molecule. Thus, wrapper (nonpolar) groups become relevant for binding interactions when an interfacial hydrogen bond relies on them in order to remain over a critical wrapping value essential on stability terms. Such a decomposition of the complex interface into a web of three-body cooperative interactions enabled us to successfully predict the hot spots reported by alanine-scanning experimental studies for a set of protein-protein complexes [15]. Additional support to this methodology comes from our recent finding that drugs disruptive of protein-protein interfaces tend to mimic the wrapping behavior of the protein they are meant to replace [16]. Such wrapping concept is precisely the kind of knowledge we exploit in the present study.

Within the above-expounded context, the aim of the present paper is two-fold. In the first place, we shall study the impact produced by the oncogenic Y220C mutations in terms of wrapping. We shall show that the tyrosine to cysteine mutation significantly lowers the wrapping content at the mutation site. Most notably, the mutation promotes the creation of a dehydron (the hydrogen bond between cysteine 220 and threonine 155) that was not present in the wild-type molecule (the corresponding hydrogen bond between tyrosine 220 and threonine 155 was well wrapped in the wild-type molecule). This dehydron is also vicinal to another dehydron preexistent in the wild-type molecule and whose wrapping content is further diminished by the mutation. Since dehydrons imply local instabilities [8], [10] and are correlated with structural disorder [12], our study allows for a rationalization of the oncogenic nature of the mutation. Indeed, our dynamical studies will show that the oncogenic mutation loosens the HB with THR 155, which breaks and reforms in the course of time, thus promoting a local structural vulnerability/destabilization. Such a wrapping decrease and the dehydron creation also explain the druggability of the mutation site, eager to receive additional stabilization by means of intermolecular wrapping. In fact, we shall see that both the PhiKan083 [7] molecule and some drug-like fragments experimentally studied [2] do in fact provide wrapping to this region. Particularly, the binding is shown to restore the wrapping content of the hydrogen bond between tyrosine 220 and threonine 155 to a value above the dehydron threshold, thus removing the local vulnerability created upon mutation and reinforcing this HB. Addtionally, we will show that the molecule PhiKan083 still leaves a flank of the CYS 220– THR 155 HB unshielded from water. Thus, modifications of the small molecule in order to also provide wrapping to such site would improve its behavior. In fact, we have shown that such a modification further stabilizes the HB and increases the binding free energy. In this regard, after completion of this work we learnt on a very recent development by the group pf Prof. Alan Fersht where they generated new small molecules based on the knowledge gained with PhiKan083 [17]–[19]. Precisely, a molecule that targets the region we indicate here by means of an acetylene linker, not only significantly increased the binding affinity as compared to PhiKan083, but showed induction of apoptosis in a human cancer cell line with homozygous Y220C mutation.

We hope the kind of analysis we present here that signals relevant drug-protein binding interactions could be of help in future efforts of drug design/(re)engineering.

## Materials and Methods

We analyzed the wrapping of the protein T-p53C studied experimentally by the group of Alan Fersht, both containing and not containing the mutation Y220C. T-p53C is a superstable version of p53 with wild-type-like properties, and the structure is virtually identical to the wild-type apart from the mutated side chains [2]. We also analyzed the wrapping of the Y220C mutated protein in complex with the small molecule PhiKan083 and with some drug-like fragments experimentally studied by the same authors [2], [7]. When we consider the T-p53C molecule, wrapping to its different HBs would come from the side chains residues of its own protein chain. In the cases of the complexes, additional wrapping would come from the nonpolar moieties/groups of the corresponding small-molecule ligand. To complete this description it is necessary to classify pairwise electrostatic interactions, HBs, in terms of an abundance distribution P(ρ), where ρ is the number of three-body correlations associated with an interaction. Thus, the extent of hydrogen-bond protection can be determined directly from atomic coordinates (calculated directly from the pdb structure of the protein or the protein-small molecule complex of interest). This parameter indicates the number of three-body correlations engaging the HB and is also known as the wrapping of the bond [9]–[15] and denoted ρ.It is given by the number of wrapper groups provided by the protein: the number of side-chain carbonaceous nonpolar groups (CH_n_, n = 0, 1, 2, 3, where the carbon atom of these groups is not bonded to an electrophilic atom or polarized group) contained within a desolvation domain around the HB. In turn, when we consider the complex between T-p53C and PhiKan083 or some of the molecular fragments, we shall also count the number of carbonaceous groups provided by the small molecule. In any case, each wrapping nonpolar group represents the third body within a three-body correlation involving the HB. The desolvation domain considered is typically defined as the reunion of two intersecting spheres of fixed radius (∼thickness of three water layers) centered at the α-carbons of the residues paired by the hydrogen bond. In structures of pdb-reported soluble proteins, HBs are protected on average by ρ = 26.6±7.5 side-chain nonpolar groups for a desolvation sphere of radius 6 Å [9]–[15]. Thus, structural deficiencies lie in the tail of the ρ-distribution, i.e. their microenvironment contains 19 or fewer nonpolar groups, so their ρ-value is below the mean ( = 26.6) minus one standard deviation ( = 7.5). While the statistics on ρ-values for HB vary with the radius, the tails of the distribution remain invariant, thus enabling a robust identification of structural deficiencies [8]–[16]. Such underprotected interactions have been called dehydrons [8]–[16], a structural motif which has been extensively discussed in the literature and identified in soluble proteins with pdb-reported structure [8]–[16]. As in [15], we only considered HBs and decided to leave aside side chain - side chain HBs from the cooperativity analysis based on the following grounds: The fluctuational nature of surface side chains imposes an entropic cost associated with hydrogen bond formation which makes the latter marginally stable at best. Also, the wrapping statistics for side chain hydrogen bonds are essentially flat with no clear distinction of the tails of the distribution do to the conformational richness of the side chains.

We also performed Molecular Dynamics (MD) simulations of a human p53 core domain mutant M133L-V203A-Y220C-N239Y-N268D (T-p53C) (pdb ID = 2J1X) placed within a box of 10182 TIP5P water molecules. We used the AMBER 11 molecular simulation suite [20] and the force field employed in the simulation was ff99SB. We also simulated the p53 core domain mutant Y220C bound to the stabilizing drug-like small-molecule PhiKan083 (pdb ID = 2VUK) placed within a box of 10065 TIP5P water molecules. The force field used in the simulation was ff99SB combined with GAFF. For the study of the complexes between the T-p53C protein and modifications of the molecule PhiKan083, we started from the original pdb 2VUK, we modified the small molecule, generated the entry parameters by means of ANTECHAMBER and solvated with the same number of TIP5P water molecules (10065) also using ff99SB and GAFF. In all cases we performed simulations at a temperature of T = 300 K, mean pressure of 1 bar, and average density around 1.0 kg*/*dm3. Equilibration was carried out in two steps: In a first step we brought the system to the corresponding temperature value with a Langevin thermostat with 0.002 ps time step and using the SHAKE algorithm; then we performed a larger equilibration within the *NpT* ensemble, also using a Langevin thermostat with 0.002 ps time step and the SHAKE algorithm. All simulations were done with AMBER 11 (PMEMD version 2.2 in GPU) and were performed in the *NpT* ensemble with a Langevin thermostat and by using a long-range-interaction cutoff of 8 Å. Equilibration was tested by monitoring the behavior of thermodynamical properties like temperature, pressure, and energy oscillations. The equilibration times were in each case much larger than the structural relaxation time (*τ_α_*, the time scale when the self-intermediate scattering function evaluated at the first peak of the structure factor has decayed to 1*/e*) for pure water at the corresponding temperature. Production runs consisted in trajectories of 2 ns. Three different replicas (different MD runs) were employed in each case.

In turn, we also performed calculations of binding free energy with AMBER by using the MM-GBSA program (Molecular Mechanics/Generalized Born Surface Area [21], [22]) with long runs of at least 2 ns. Within this formalism, the value of the free energy of binding (ΔG_bind_) of each compound was calculated according to the equation:
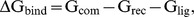
where com, rec, and lig stand for complex, receptor and ligand, respectively. The free energy of each of these was estimated as a sum of the following three terms:



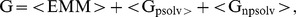
where EMM is the molecular mechanics energy of the molecule expressed as the sum of the internal energy of the molecule plus the electrostatics and van der Waals interactions, G_psolv_ is the polar contribution to the solvation energy of the molecule, G_npsolv_ is the nonpolar solvation energy. We should keep in mind that the result does not equal the real binding free energy since we did not estimate the (dis-favourable) entropy contribution to binding. Nevertheless this result could be used to compare similar systems. The snapshots for MM-GBSA analyses were taken every 10 ps of a 2000 ps MD production run, resulting in a total of two hundred snapshots. In principle, the calculation of the binding free energy described above would require three independent MD simulations of the complex and both individual proteins. However, we use the “single trajectory approach” which makes the approximation that no significant conformational changes occur upon binding so that the snapshots for all three species can be obtained from a single trajectory.

The energies were obtained using the mm_pbsa module of Amber 11. The internal, electrostatics and van der Waals energies were calculated with the sander module. The polar solvation free energies (G_psolv_) were calculated by the generalized Born (GB) approach [23], [24] implemented in Amber 11. Finally, energy estimations with GBSA were made with the Onufriev's GB parameters (igb = 2). All calculations used a solute internal dielectric constant equal to 1 and a dielectric constant of 80 for the exterior of the molecule. The nonpolar solvation term (G_npsolv_) was calculated from the solvent-accessible surface area (SASA) [25] using the equation:
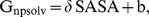
where SASA was determined with the Molsurf [25] method using a probe radius of 1.4 Å. Parameters were δ = 0.0072 kcal mol^–1^ Å^–2^ and b = 0 kcal mol^–1^ to be used with Amber PB or GB polar solvation energies.

## Results and Discussion

Table 1 shows the results of our wrapping analysis. The T-p53C molecule is labeled as chain A-wt, the T-p53C with the oncogenic mutation is denoted as chain A and the small-molecule PhiKan083 is labeled as molecule D. Thus ρ_A-wt_ would be the wrapping contributed to the HBs of T-p53C by its own protein chain A, ρ_A_ will be the corresponding case for the molecule T-p53C-Y220C (the version with the oncogenic mutation Y220C) and ρ_AD_ will be the wrapping of the HBs of T-p53C-Y220C within the complex with PhiKan083 (with wrapping contributions both from the protein chain and the small molecule).

**Table 1:** Wrapping study of the T-p53C protein molecule (pdb: 1UOL), the T-p53C-Y220C protein (pdb: 2J1X) and the complex between p53C-Y220C and the small molecule PhiKan083 (pdb: 2VUK).

HB	ρ_A-wt_	ρ_A_	ρ_AD_
Arg 110– Trp 146	24	24	28
Leu 145– Thr 230	18	17	19
Tyr/Cys 220– Thr 155	23	18	20

The wrapping value of the HBs of the protein chain that occur at the mutation site (mutation Y220C) and that are targeted by PhiKan083 is indicated respectively as ρ_A-wt_, ρ_A_ and ρ_AD_.

The first result that clearly transpires from this Table is the fact that the mutation Y220C that creates a cavity at the protein surface leads the wrapping value of the HB between TYR 220 and THR 155 (well wrapped in T-p53C, with a ρ_A-wt_ = 23) to below the dehydron threshold (ρ_A_ = 18 for the corresponding HB between CYS 220 and THR 155 in T-p53C-Y220C). Thus, the oncogenic mutation implies the creation of a dehydron in the protein chain. Moreover, we can see that the HB between LEU 145 and THR 230 (vicinal to the mutation site and already a dehydron in T-p53-Y220C) also lowers it wrapping content upon mutation Y220C. The creation of a new vulnerability site (a dehydron) and the overall wrapping content decrease should imply a local destabilization [9], [11] of the chain and an increase in local disorder [13]. Thus, the effect of the oncogenic mutation that destabilizes p53 can be accounted for in terms of the changes it produces in wrapping interactions.

In turn, the binding of the small molecule PhiKan083 restores function by stabilizing the p53 protein. From Table 1 we can clearly see that the molecule binds to the protein precisely at the dehydron sites by contributing wrapping to both dehydrons. Moreover, the drug-like molecule restores the CYS 220– THR 155 HB to a wrapping value above the dehydron threshold, thus removing the dehydron created upon the oncogenic mutation.

We have also studied the complexes between T-p53C and some of the fragments probed experimentally by Fersht’s group [2] (for example, the fragments termed as Fragment 15 and Fragment 2, which were found to bind close to the position occupied by PhiKan083 in the complex), and we found that they also tend to contribute wrapping to the dehydron created upon the oncogenic mutation (data not shown).

**Figure 1:**
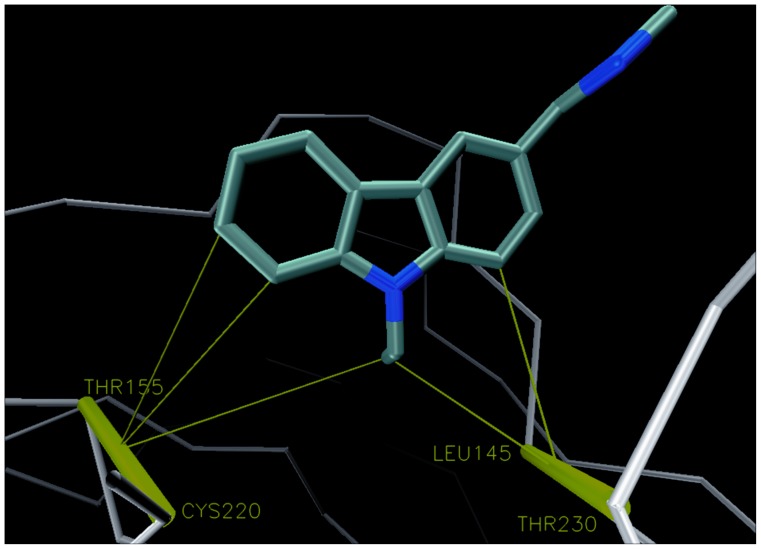
T-p53C-Y220C in complex with PhiKan083 (pdb: 2VUK). Dehydrons in the protein are depicted by green bars joining the two residues paired by the HB, while green thin lines are wrapping interactions with the small molecule.

Fig. 1 shows the complex between T-p53C-Y220C and PhiKan083 at the Y220C mutation. In the plot we indicate the wrapping three-body interactions as lines that go from the carbonaceous groups of the small molecule to the corresponding HB of the protein. We can see that PhiKan083 provides wrapping to the dehydron CYS 220– THR 155 from one side. In the study of the group of Alan Fersht [2] they found that some fragments (like Fragment 15) were placed a bit deeper within the cavity, closer to the other side of such HB. They also found that isopropanol molecules tend to place their carbonaceous groups close to such region [2]. Thus, even when PhiKan083 raises the wrapping level of the HB above the dehydron threshold, the HB still presents a flank unshielded from water attack which is located towards the other side or entrance of the cavity created upon mutation. It seems reasonable to envision a modification of the drug-like molecule by attaching to it a wrapper functional group at such end in order to better protect this HB from the disrupting effect of hydration. Additionally, another modification could include a group that further increased the wrapping level of the other dehydron of the protein at the cavity, the LEU 145– THR 230 HB.

In order to gain a better understanding of the destabilizing nature of the oncogenic mutation Y220C and of the role of the binding molecules we performed Molecular Dynamics (MD) simulations.

**Figure 2:**
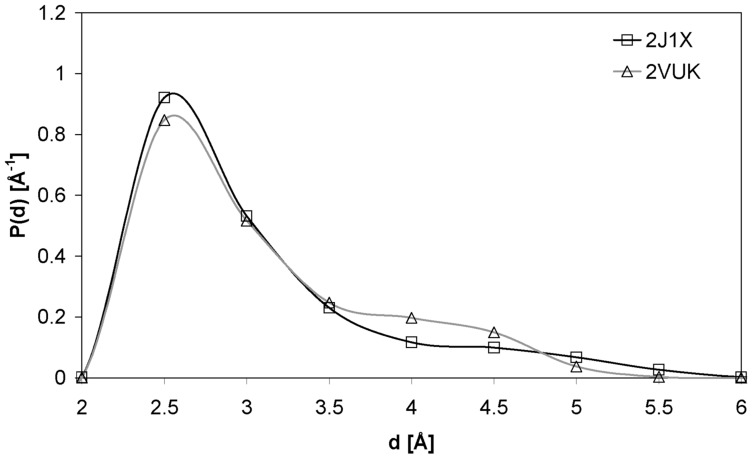
Distribution of the distance between the C = O oxygen of THR 155 and the oxygen of the nearest water molecule. We show this curve for pdb 2VUK (T-p53C-Y220C-PhiKan083; mean value of the distribution μ = 3.102 Å and deviation σ = 0.692 Å) and 2J1X (T-p53C-Y220C; μ = 3.065 Å and σ = 0.736 Å). In both cases we used 2000 configurations and water was modeled with the TIP5P model.

Fig. 2 shows the distribution of the distance (d) between the carbonylic oxygen of THR 155 and the nearest water molecule. We recall that THR 155 forms a HB with CYS 220 in the mutated T-p53C-Y220C (pdb 2J1X, free protein) and in the protein bound to PhiKan083 (2VUK). In the second case we are dealing with a well wrapped HB while in the first case this HB constitutes a dehydron. From this picture it is evident that the water molecules get a bit closer to the dehydron of the protein (free protein, 2J1X) so as to attack the intramolecular interaction. In fact, the corresponding peak is compatible with a water-protein hydrogen bond formation when water acts as a donor [26].

**Figure 3:**
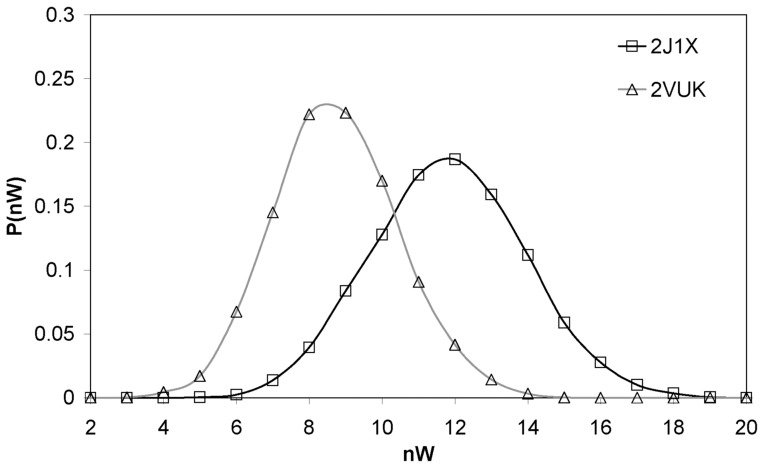
Distribution of the number of water molecules within the desolvation domain of the HB CYS 220 - THR 155. Pdbs 2VUK (T-p53C-Y220C-PhiKan083; μ = 8.743 and σ = 1.720) and 2J1X (T-p53C-Y220C; μ = 11.819 and σ = 2.111).

Furthermore, in Fig. 3 we show the distribution of the number of water molecules (nW) within the desolvation domain of the HB CYS 220 - THR 155 (as already indicated, this domain is built as a pair of spheres of radius 6 Å, centered at the α carbons of both residues involved in the HB). As expected, the dehydron CYS 220 - THR 155 in the free protein shows a higher water population than the well-wrapped CYS 220– THR 155 HB in the PhiKan083 bound protein.

**Figure 4:**
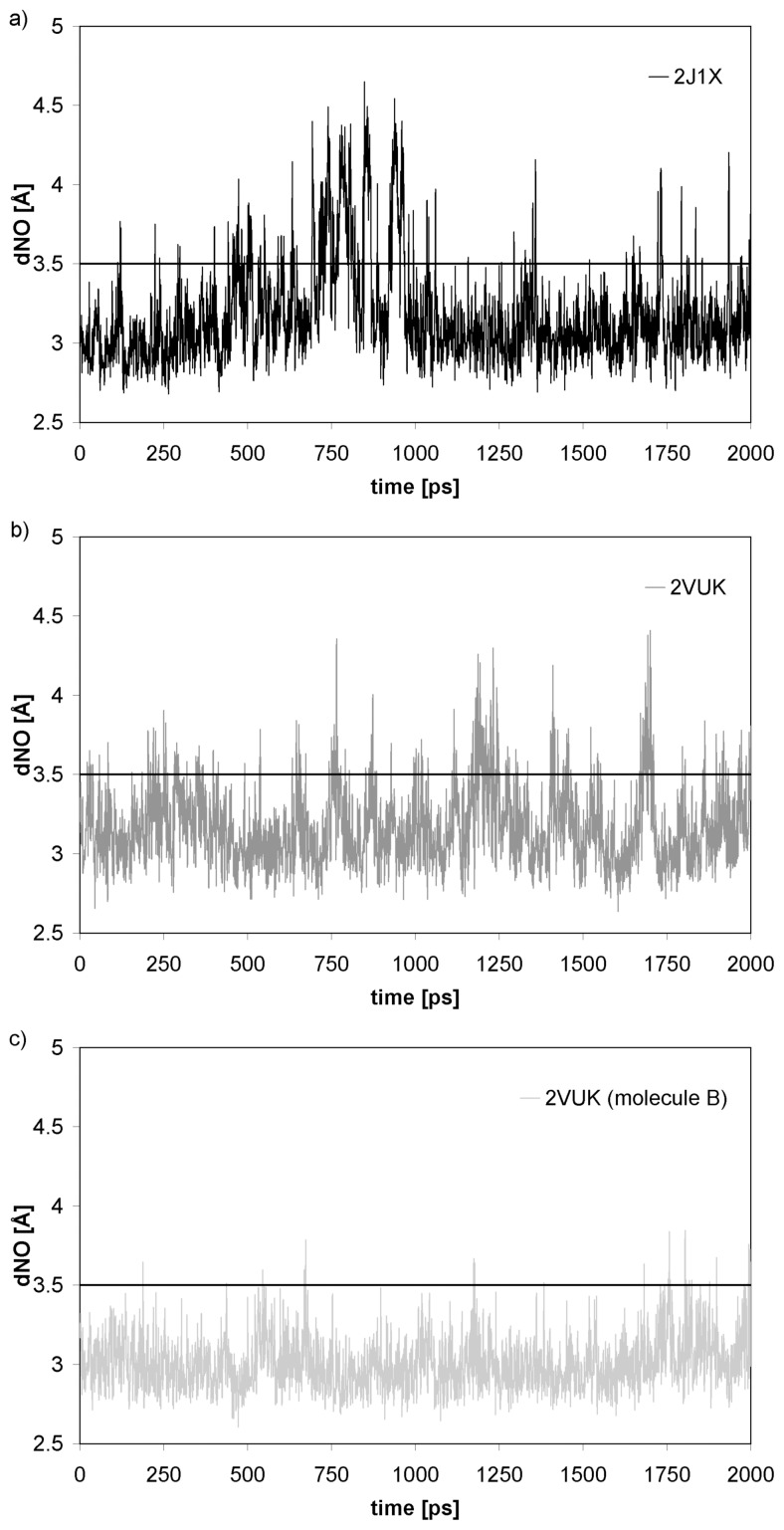
Time evolution of the distance between the backbone amide nitrogen atom of CYS 220 and the backbone carbonylic oxygen of THR 155. Simulations form pdbs 2J1X (Fig. 4 a; μ = 3.169 and σ = 0.323) and 2VUK (Fig. 4 b; μ = 3.165 and σ = 0.254). A cutoff of 3.5 Å for the hydrogen bond was chosen. We have also included (Fig. 4 c; μ = 3.003 and σ = 0.173) the case of molecule B (a modification of PhiKan083, please see below) in complex with T-p53C-Y220C, as simulated form a starting configuration generated from a modification of pdb 2VUK. We mention that this graph shows a single MD run for each case.

**Figure 5:**
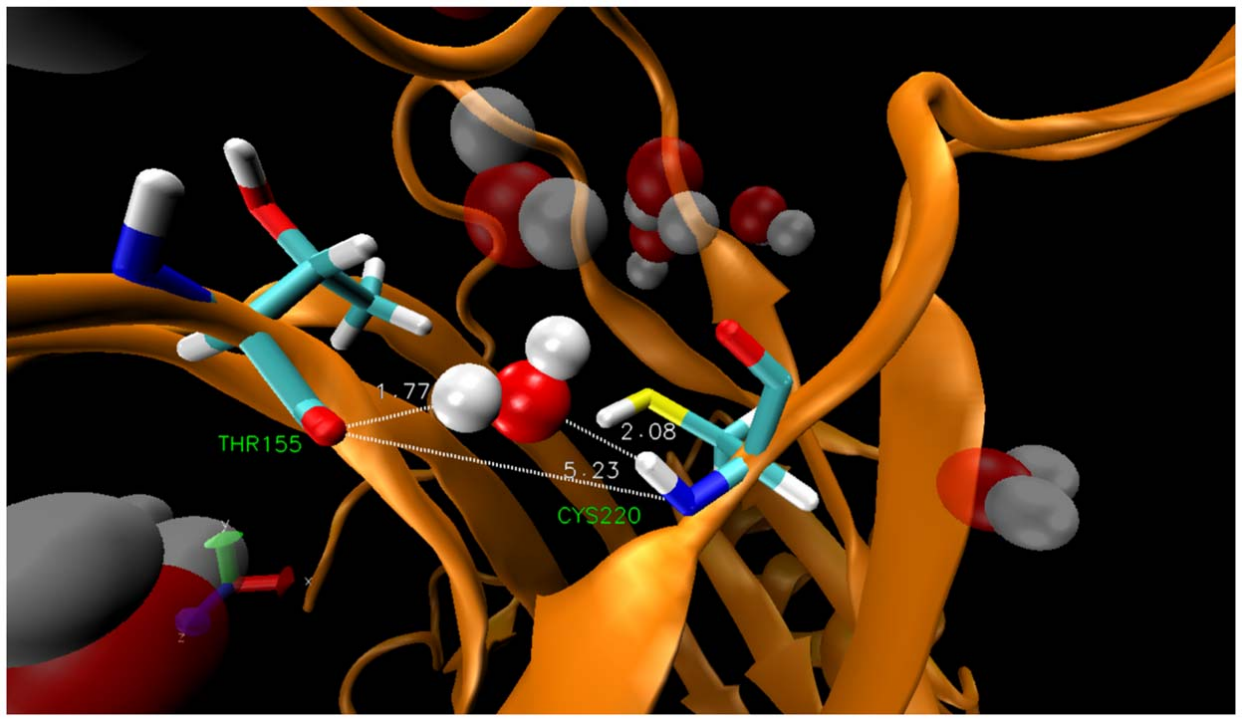
An instance of intercalation of a water molecule between THR 155 and CYS 220 in 2J1X.

In turn, Fig. 4 exhibits the time evolution of the distance between the backbone amide nitrogen atom of CYS 220 and the backbone carbonylic oxygen of THR 155 (dNO) along a simulation (we considered 2000 configurations within a total simulation time of 2 ns). We can observe that the fluctuations are clearly more significant for the case of 2J1X protein (the free protein with the oncogenic mutation). In fact, the HB breaks (surpasses the HB distance threshold of 3.5 Å) several times in the interval that goes from 500 ps to 1000 ps (also take into account the σ values in the caption of Fig. 4). The dehydron creation upon mutation thus promotes a local loosening in the protein chain since an intermolecular interaction (the HB studied) gets destabilized with temporal loss of local structural elements (the CYS 220– THR 155 HB represents an unstable intramolecular contact which is easily disrupted by water). Thus, our results provide a further support to the oncogenic nature of the Y220C mutation that destabilizes the protein and lowers its melting temperature. In some trajectories one can even find that at certain time a hydration water molecule bridges the HB CYS 220– THR 155 interaction (intercalating between both partners) and forming a couple of new HBs: O–H-N with CYS 220 and C = O–H-O with THR 155. This situation is illustrated in Fig. 5 and lasts many configurations until the intramolecular HB is re-established.

On the other hand, in the presence of the molecule PhiKan083 (complex 2VUK), which provides wrapping to the dehydronic HB CYS 220– THR 155, the N-O distance is a bit more stable during the whole simulation, fluctuating more smoothly around a mean value which is much lower than the HB threshold value. This fact rationalizes the behavior of the small molecule which stabilizes the protein and raises its melting temperature, thus restoring function.

It is also worth noting that the simulations show that the water molecules approach the CYS 220– THR 155 dehydron from the side of the protein cavity not occupied by PhiKan083. Thus, the wrapping provided by such small molecule is very anisotropic. Even when the HB increases its wrapping to a value above the dehydron threshold (thus, the PhiKan083 molecule would remove the dehydron if one merely considered the arithmetic ρ-value) it lefts a flank completely unprotected at the other side of the cavity. Water molecules could penetrate the cavity from such side and attack the intramolecular HB. In fact, in Fig. 4 we can see that the N-O distance of such HB indeed fluctuates and eventually slightly surmounts the HB threshold value (3.5 Å). In such figure we have also included the case for our proposed modification of PhiKan083 (molecule B, where that wrapping of the HB increases even more than with PhiKan083, please see below), which indeed performs much better. It is worth noting that the standard deviation for the distributions falls as the wrapping of the HB increases, that is, as we go from Fig. 4a. to Fig. 4c.

**Figure 6:**
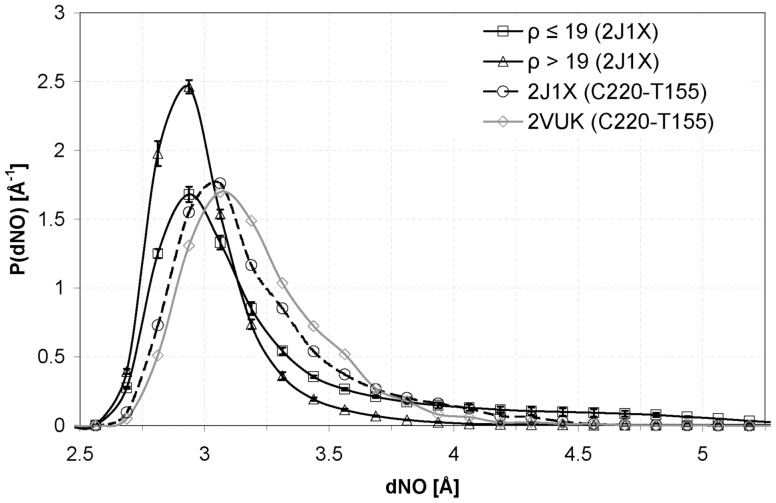
Distribution for the distance between the amidic N atom and the carbonylic O atom. HBs with ρ>19 (well wrapped) in 2J1X (μ = 2.949 and σ = 0.276), HBs with ρ≤19 (poorly wrapped) in 2J1X (μ = 3.208 and σ = 0.564), CYS 220– THR 155 in 2VUK (μ = 3.150 and σ = 0.288) and CYS 220– THR 155 in 2J1X (μ = 3.143 and σ = 0.339). Attention should be paid to the tails of the distributions, that is, at large values of the N-O distance. These data come from the study of the three replicas for each case.

To make this observation more precise, In Fig. 6 we show the distribution of the CYS 220– THR 155 N-O distance (dNO) during the simulation. We show the results for the free protein molecule and the case for the complex with PhiKan083. Again, this graph shows that the small molecule makes the HB more stable by shortening the distance for the HB partners (please see the tails of the distributions, that is, the behavior at large values of the N-O distance which shows that the curve for the complex with PhiKan083, 2VUK, decays earlier than that for the free protein, 2J1X). In such graph we have also included the average behavior of the different HBs of the protein, both dehydrons (ρ≤19) and non-dehydrons or well wrapped HBs (ρ>19). We can easily see that the dehydrons present a large tail in the range above the HB threshold value while the curve for the well-wrapped HBs decays much earlier. Thus, the CYS 220– THR 155 HB of the complex between PhiKan083 and the protein displays a behavior that is somehow intermediate between these two limit cases. This reinforces the idea that the protection provided by the small molecule is only partial given the fact that it leaves a flank unshielded. Again, from Fig. 6 we can learn that the standard deviation of the distributions falls as the wrapping of the HB increases.

**Figure 7:**
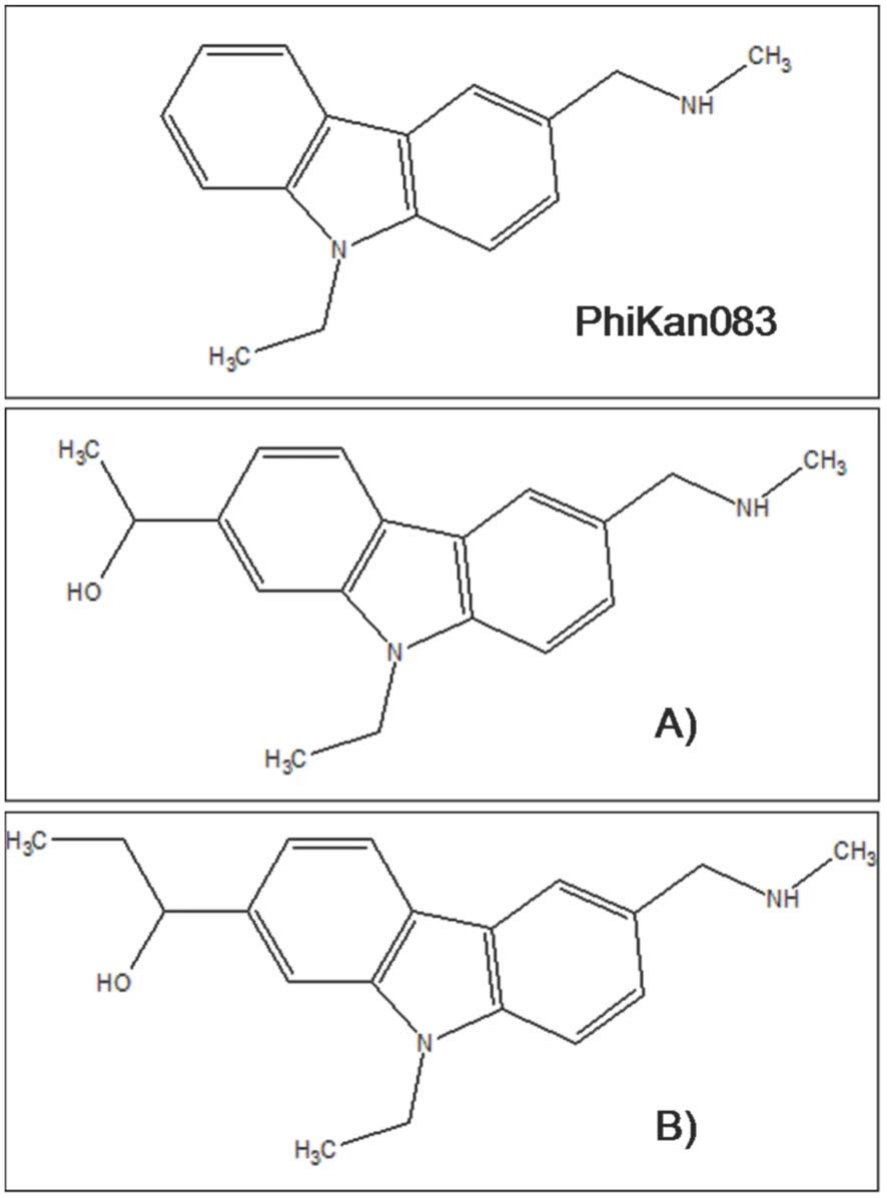
Molecular structure and of Phikan083 and modifications. The mean value we calculate for the binding free energy of the complex T-p53C-Y220C-PhiKan083 (2VUK) is <ΔG_binding_> = −29.42 kcal/mol (Standard deviation, STD = 2.05 kcal/mol). A) First modification of Phikan083. <ΔG_binding_> = −29.80 kcal/mol (STD = 2.71 kcal/mol). B) Second modification of Phikan083. <ΔG_binding_> = −35.33 kcal/mol (STD = 2.81 kcal/mol).

These facts point to the possibility of a rational redesign or improvement of the small molecule PhiKan083. Thus, we proposed to modify such small molecule by attaching some wrapper functional groups that might improve the wrapping protection of the CYS 220– THR 155 HB (see Fig. 7). Thus, the new small molecule would also reach the other side of the cavity. From the results of the free energy of binding (please see later on for the details of the calculations) we chose the second modification (case B, the molecule which provides the better shielding from water) for further studies. Thus, by replacing the PhiKan083 by this molecule (case B) within the 2VUK pdb, we performed new MD simulations with AMBER.

**Figure 8:**
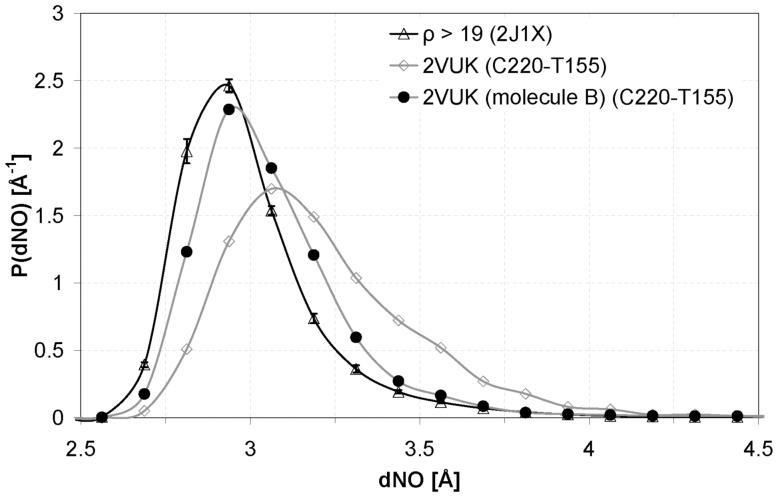
Distribution of the distance between the N atom of the amide group and the O atom of the carbonyl. HBs with ρ>19 in 2J1X (μ = 2.949 and σ = 0.276), CYS 220– THR 155 in 2VUK (μ = 3.150 and σ = 0.288) and CYS 220– THR 155 in the second modification of Phikan083 (molecule B in Fig. 7; μ = 3.000 and σ = 0.235).

**Figure 9:**
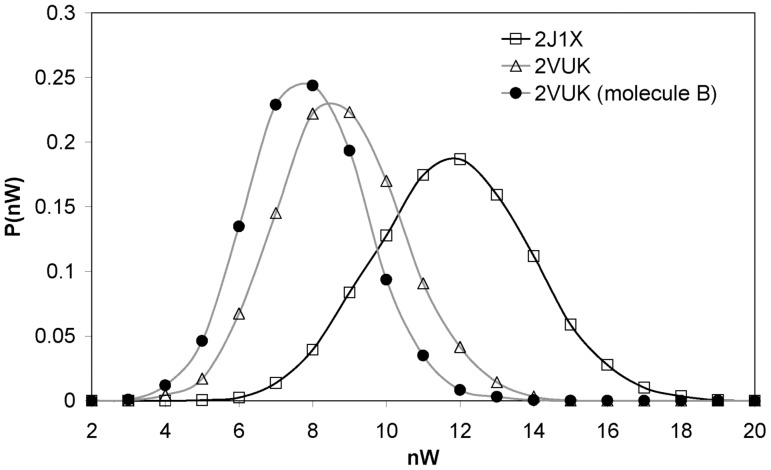
Distribution of the number of water molecules within the desolvation domain of the HB CYS 220– THR 155. 2J1X (μ = 11.819 and σ = 2.111), 2VUK (μ = 8.743 and σ = 1.720) and CYS 220– THR 155 in the second modification of Phikan083 (molecule B, μ = 7.847 and σ = 1.573).

Figure 8 shows the improvement this new molecule produces in the CYS 220– THR 155 HB distance. The new molecule, which better wraps the HB, indeed yields a distribution very similar to the one corresponding to well wrapped HBs of 2J1X, thus making the HB even much more stable than PhiKan083 (lower mean value and deviation). This strengthening of the CYS 220– THR 155 HB is also evident in Fig. 4c which tells us that, at variance from the situation in the free protein or in the case of the complex with PhiKan083, the N–O distance remains practically all the simulation time below the HB threshold value. Additionally, in Fig. 9 we show a plot of the number of water molecules within the desolvation domain of CYS 220– THR 155 HB. We show the case for the free oncogenic T-p53C-Y220C, for the complex with PhiKan083 and for the complex with the new molecule (molecule B in Fig. 7).

**Figure 10:**
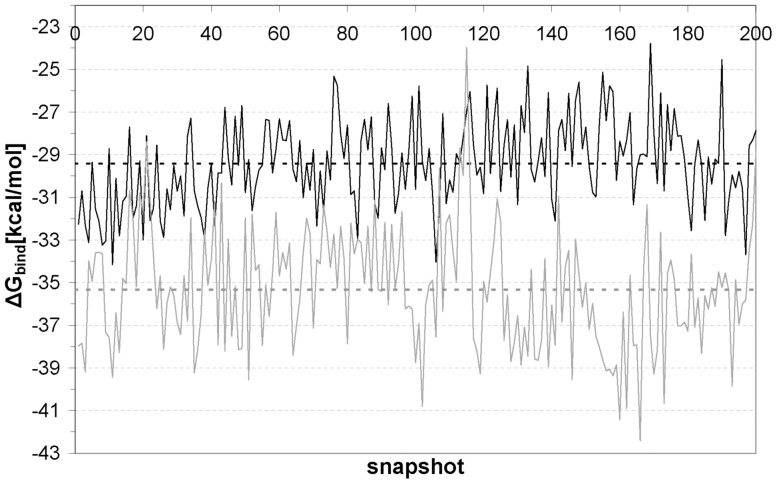
Time evolution for the binding free energy (ΔG_binding_) during a simulation run. Black line: T-p53C-Y220C-PhiKan083; gray line: 2nd modification of PhiKan083 (B) – T-p53C-Y220C; dotted black line: mean value for T-p53C-Y220C-PhiKan083; dotted gray line: mean value for the 2nd modification of PhiKan083 (B) – T-p53C-Y220C.

In turn, we performed a calculation of the binding free energy with AMBER by using the MM-GBSA program (Molecular Mechanics/Generalized Born Surface Area). Our results are displayed in Fig. 10. We can see that the new molecule produces a greater ΔG of binding to T-p53C-Y220C. While the mean value for PhiKan083 is roughly 29 Kcal/mol, the modified molecule yields a value of around 35 Kcal/mol. Thus, these results point to the feasibility of an improvement of lead compounds by considering the wrapping interactions. In the modified PhiKan083 case, the protein structure is strengthened by tying or fastening the HB responsible for the oncogenic mutation, given the fact that this intramolecular interaction is now well shielded from water attack.

**Figure 11:**
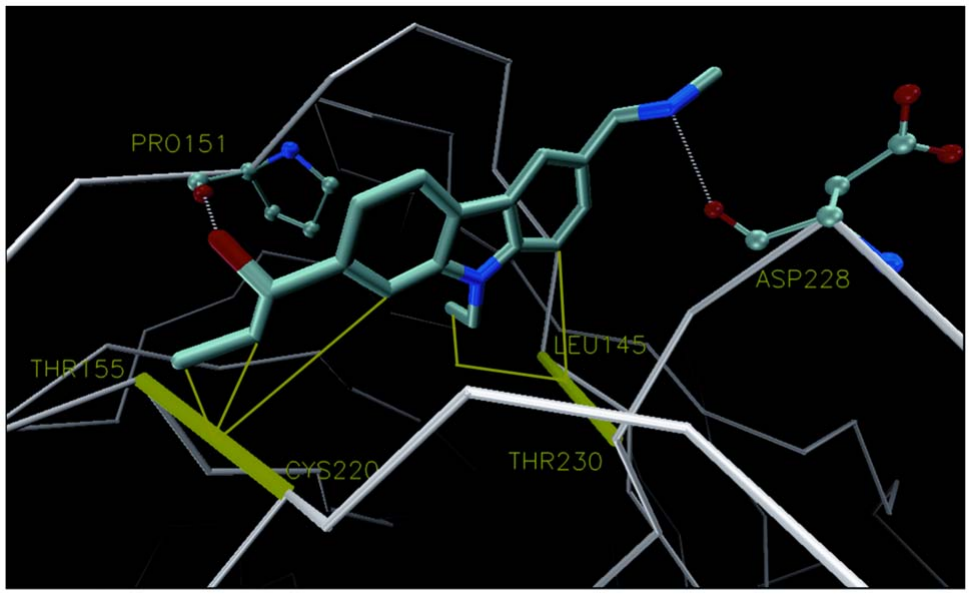
Illustration of the wrapping interactions between T-p53C-Y220C and the second modification of PhiKan083 (molecule B in **Fig.7**).

Finally, Figure 11 illustrates the wrapping interactions in the complexes of T-p53C-Y220C with the second modification of PhiKan083 (molecule B in Fig.7). The modified small-molecule penetrates deeper into the cavity thus providing a better shielding from water to the CYS 220– THR 155 HB and forms an additional HB with PRO 151. Interestingly, the study of Fersht’s group found an enhanced isopropanol density around this latter place when simulating the protein in a mixture of water-isopropanol. Additionally, after our work was finished we learnt that in a very recent work this group developed new small molecules which extended the region targeted by PhiKan083 in both extremes [17]. Precisely, only the extension of the lead molecule to target the region we indicate here by means of an acetylene linker provided a significantly increased binding affinity to the low µM range and a substantial stabilization of the protein as compared to PhiKan083. Such small molecules, called PhiKan5174 and PhiKan5196, also produced induction of apoptosis in a human cancer cell line with homozygous Y220C mutation. In addition, these compounds have been used to highlight the fact that p53-Y220C aggregation is modified significantly [18], [19].

### Conclusion

In summary, our study has revealed that the oncogenic Y220C mutation that lowers the stability of the tumor suppressor protein p53 can be explained by the reduction it produces in the wrapping content at the mutation site and, more significantly, by the creation of a net structural vulnerability, a dehydron. This mutation locally loosens the protein structure since the dehydronic CYS 220– THR 155 HB is now accessible by water and wildly fluctuates, breaking and reforming in the course of time, thus promoting the structural destabilization. The binding of drug-like small molecules intended to rescue the function of p53 can be explained precisely by the wrapping such molecules contribute to the mutation site. We show that the small molecule in fact removes the dehydron created upon the oncogenic mutation. Thus, such molecules tighten the CYS 220– THR 155 HB and locally stabilize the protein structure. However, some function-rescue molecules (like PhiKan083) only provide wrapping from one side of the cavity where such HB is placed, entirely leaving the other flank unshielded. Thus, we have shown that a modification of such molecule better wraps the HB and further strengthens the protein chain at the local site. We also show computationally that such a modified molecule presents an increased binding free energy as compared with PhiKan083. We expect this knowledge could be instrumental in the re-engineering of such kind of molecules to gain a better affinity, in the rational design of new drugs for this case, and also to help in other cases of drug design/(re)engineering.
